# Unipolar Signal Augmentation as a Novel Marker of Tissue Contact in Pulsed Field Ablation

**DOI:** 10.1002/joa3.70168

**Published:** 2025-08-07

**Authors:** Yasuyuki Takada, Junichi Kamoshida, Muryo Terasawa, Kazuhiro Satomi, Yoshinao Yazaki

**Affiliations:** ^1^ Department of Cardiology Tokyo Medical University Tokyo Japan

**Keywords:** atrial fibrillation, catheter ablation, contact force, unipolar signal, pulsed field ablation

## Abstract

A positive deflection following the atrial P wave in unipolar electrograms serves as a practical surrogate marker for catheter‐tissue contact during pulsed field ablation; correlating with effective lesion formation across diverse anatomical regions.

## Graphical abstract

Unipolar electrograms exhibited a post‐P‐wave positive deflection at contact sites verified by intracardiac echocardiography (ICE). Notably, this signal persisted after ablation and corresponded to effective lesion formation, supporting its value as a practical marker for assessing tissue contact during and after pulsed field ablation (PFA).

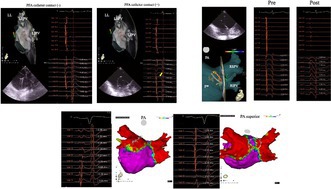

Pulsed field ablation (PFA) has been established as an effective and tissue‐selective ablation for atrial fibrillation (AF) ablation, demonstrating favorable safety and efficacy profiles in both paroxysmal and persistent AF populations [1]. Despite its unique nonthermal mechanism, lesion formation in PFA is still influenced by tissue contact conditions. Recent work suggests that sufficient proximity between the catheter and atrial wall improves lesion depth and durability, even under pulsed‐field energy application [2]. However, most PFA systems lack dedicated contact force sensors or impedance monitoring. Therefore, identifying reliable, real‐time surrogate markers of contact remains clinically relevant.

In thermal ablation, unipolar electrograms are known to exhibit morphological changes such as post‐P‐wave deflections and ST‐segment shifts, reflecting mechanical interaction with the endocardium [3]. Similar features may be observable during PFA and used to infer catheter‐tissue contact. We describe three cases in which a positive deflection following the atrial P wave in unipolar signals was associated with effective lesion formation. This observation may support the use of unipolar morphology as a practical indicator of catheter‐tissue apposition in PFA procedures.

## 1 Case 1

**FIGURE 1:**
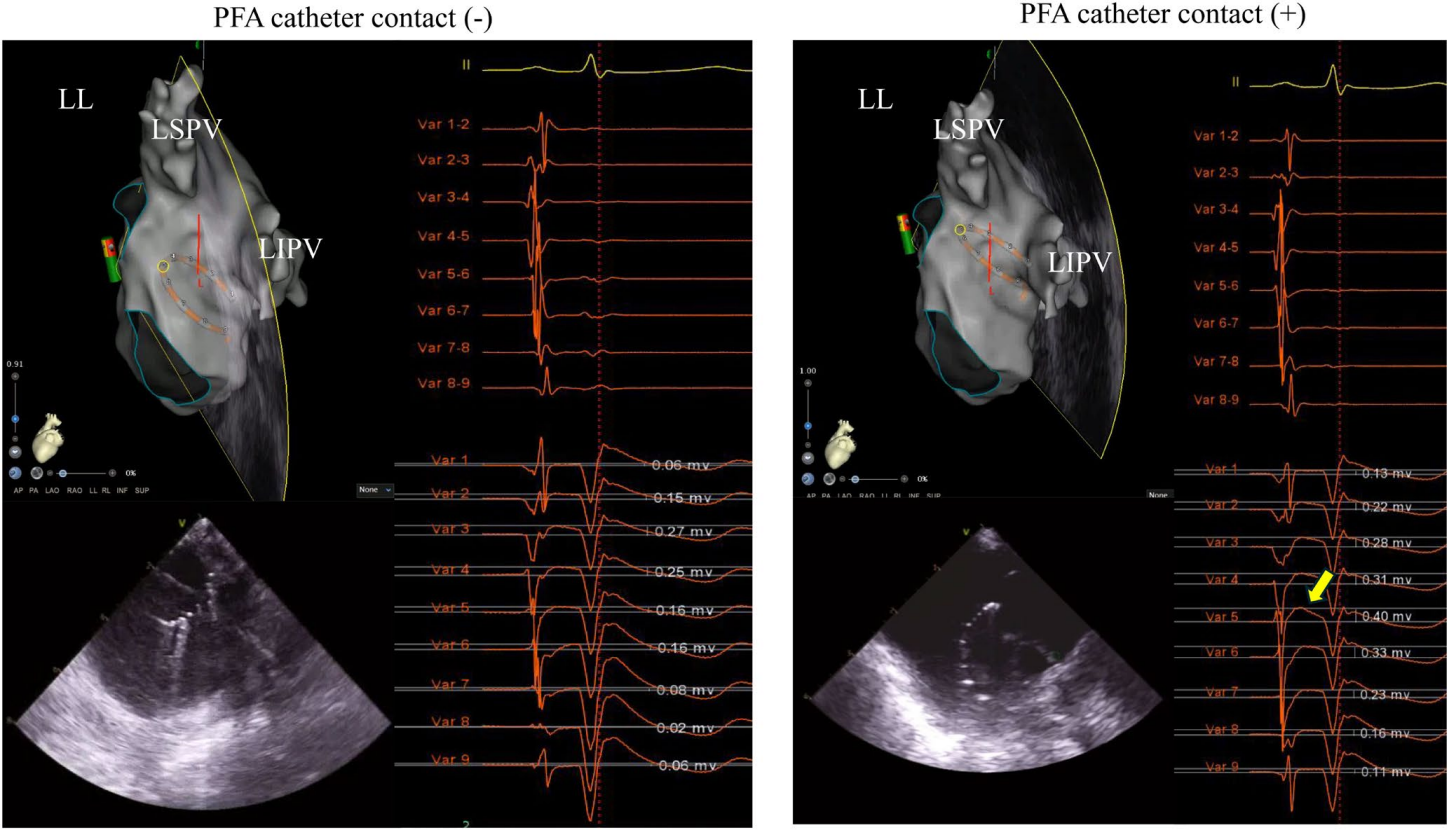
Myocardial contact of the pulsed field ablation (PFA) catheter was assessed using intracardiac echocardiography (ICE) and 3D electroanatomical mapping. In the absence of contact, both bipolar and unipolar recordings showed local potentials, but post‐P‐wave positive deflections were minimal. In contrast, when contact was confirmed, no significant change was observed in local electrograms, yet a marked increase in post‐P‐wave positive deflection was evident (yellow arrows). The contact site, located at the left pulmonary vein ridge, is indicated by yellow dots.

**FIGURE 2:**
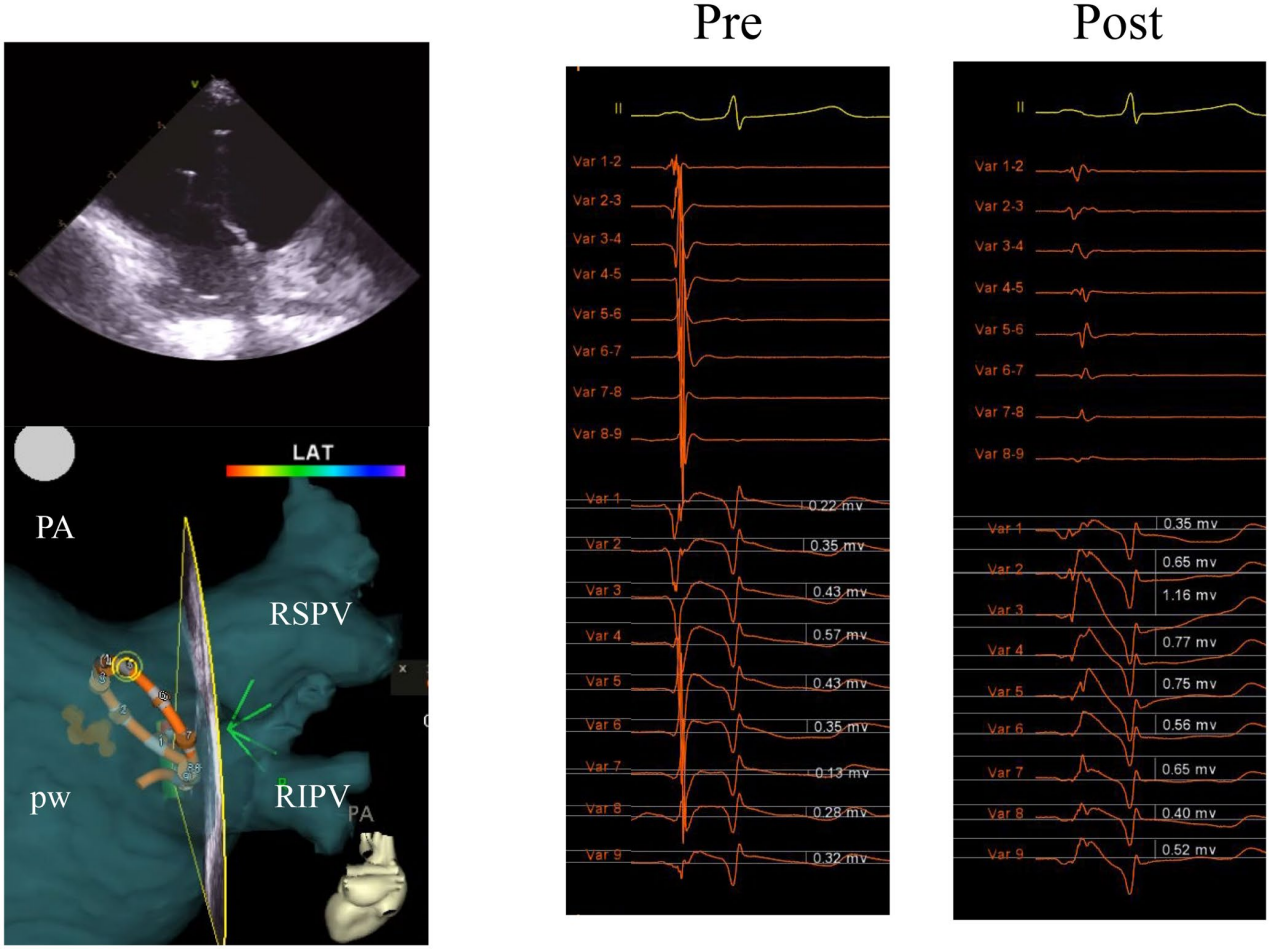
Pulsed field ablation (PFA) applied to the anterior aspect of the right superior pulmonary vein. Electrodes 4 and 5, confirmed to be in contact with myocardial tissue by intracardiac echocardiography, are highlighted as small circles on the 3D map. These electrodes demonstrated higher post‐P‐wave positive deflections on unipolar recordings compared to adjacent electrodes. Although bipolar signals disappeared after ablation, the post‐P‐wave positive deflections persisted in the unipolar electrograms.

A 60‐year‐old man with dilated cardiomyopathy and paroxysmal AF underwent pulmonary vein isolation (PVI) using the PulseSelect catheter (Medtronic) and the CARTO mapping system. Unipolar electrograms at contact sites consistently exhibited a positive deflection immediately after the atrial P wave. These findings correlated with good contact as assessed by intracardiac echocardiography (ICE), and were not observed in areas with poor apposition (Figure 1). Bipolar local potentials decreased before and after ablation, but the unipolar positive deflection remained (Figure 2).

## 2 Case 2

**FIGURE 3:**
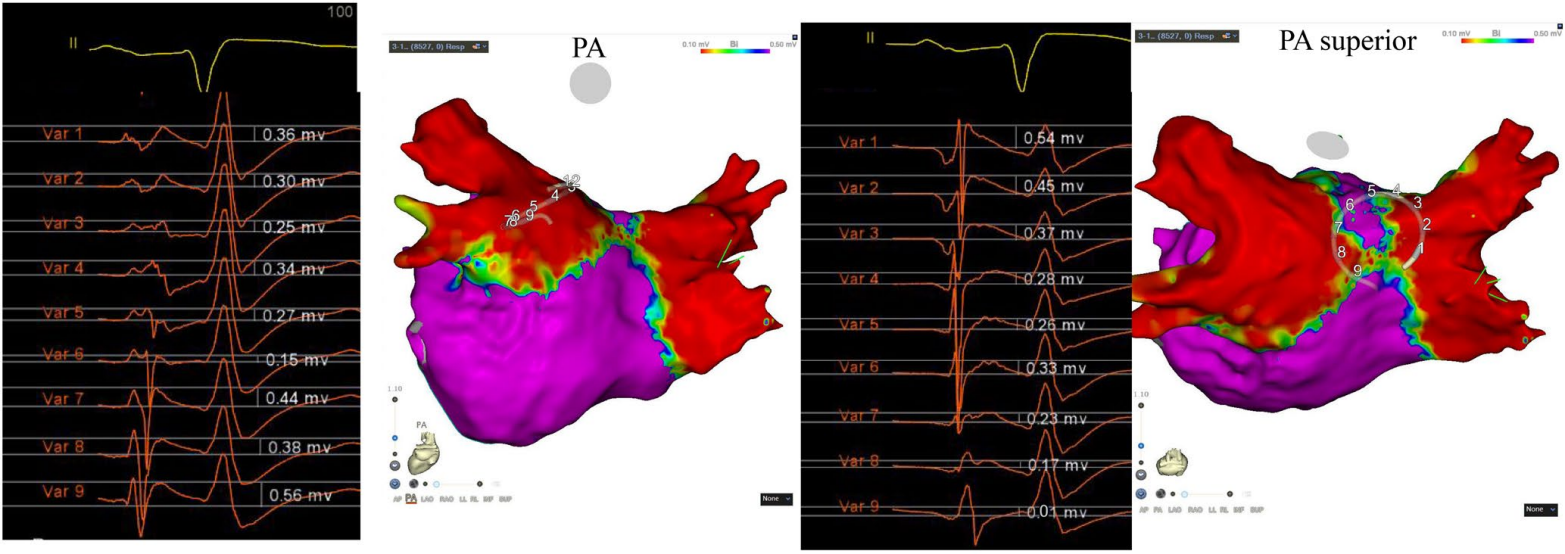
Left: Post‐ablation voltage map of the pulmonary veins. At sites where pulsed field ablation (PFA) was delivered, all electrodes of the ablation catheter exhibited a positive deflection following the P wave on unipolar electrograms, indicating adequate tissue contact. Corresponding scar formation was confirmed on voltage mapping. Right: Post‐ablation voltage map of the left atrial roof. A residual conduction gap is evident at the site where unipolar electrograms lacked a post‐P‐wave positive deflection, suggesting suboptimal catheter‐tissue contact during PFA.

A 75‐year‐old man with paroxysmal AF and no structural heart disease underwent PVI and roof line ablation using the same catheter system. During pulmonary vein ablation, a positive deflection after the atrial P wave was observed in unipolar signals at sites that later demonstrated scar formation on post‐ablation voltage mapping. In contrast, a residual gap on the left atrial roof lacked this feature, and voltage mapping revealed preserved potentials at this location (Figure 3).

## 3 Case 3

**FIGURE 4:**
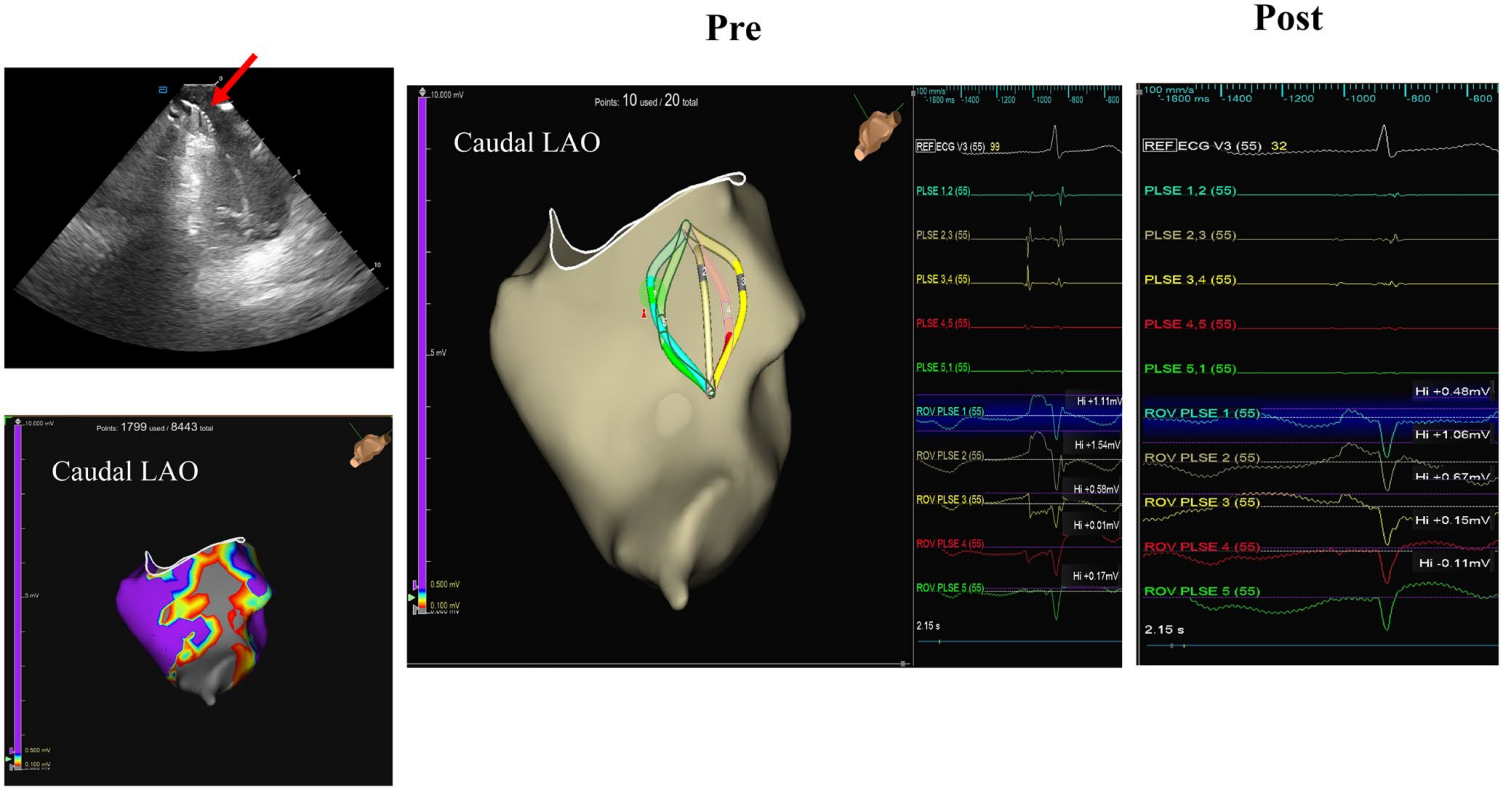
Tissue contact of the pulsed field ablation (PFA) catheter was confirmed by intracardiac echocardiography. The catheter was positioned along the cavotricuspid isthmus (CTI) line on 3D mapping, and unipolar electrograms from electrodes 1, 2, and 3 exhibited post‐P‐wave positive deflections. Bipolar electrograms demonstrated signal elimination after ablation, while the post‐P‐wave positive deflection persisted in unipolar recordings. The post‐ablation voltage map showed complete scar formation along the CTI line.

A 75‐year‐old woman with paroxysmal AF and typical atrial flutter underwent PVI and cavotricuspid isthmus (CTI) ablation using the FaraPulse catheter (Boston Scientific) and the EnSite NavX system. Before ablation, a clear post‐P‐wave positive deflection was seen in unipolar electrograms, which corresponded to ICE‐confirmed tissue contact. Following ablation, voltage mapping showed a low‐voltage line across the CTI consistent with successful bidirectional block (Figure 4).

## 4 Discussion

These cases suggest that a positive deflection following the atrial P wave in unipolar electrograms may serve as a practical surrogate marker for catheter‐tissue contact during pulsed field ablation. This feature was observed across diverse anatomical regions and mapping systems. While bipolar electrograms also reflect local tissue signals, their amplitude typically diminishes after ablation, limiting post‐treatment contact assessment. In contrast, unipolar recordings may retain characteristic deflections even after energy delivery, enabling continued evaluation of catheter position relative to the endocardium.

This distinction may be particularly valuable in identification of which specific electrodes among the multiple electrodes of the pulsed field catheter are in contact with tissue, potentially serving as a useful predictor of treatment efficacy. In our case series, sites demonstrating clear post‐P‐wave positive deflections consistently correlated with electrogram elimination on post‐ablation voltage mapping, suggesting high sensitivity for effective lesion formation. Conversely, areas lacking this unipolar feature, such as the residual gap observed on the left atrial roof in Case 2, showed preserved potentials on voltage mapping, indicating incomplete ablation. This observation supports both the sensitivity and positive predictive value of this surrogate marker; though systematic evaluation with larger cohorts is required to establish definitive thresholds.

Regarding anatomical considerations, even in regions prone to far‐field signals such as the ridge between pulmonary veins and the left atrial appendage, the post‐P‐wave positive deflection remained clearly distinguishable from background electrical activity when tissue contact was confirmed by intracardiac echocardiography. This observation suggests that the mechanical component of catheter‐tissue interaction generates a distinct electrogram signature that differs from purely electrical far‐field phenomena. The morphological characteristics of this deflection appear to be sufficiently robust to overcome potential interference from distant cardiac structures.

While this proposed unipolar signal assessment may facilitate optimal catheter‐tissue contact for enhanced lesion efficacy, maintaining procedural safety remains paramount. Previous studies have demonstrated that contact force‐guided catheter ablation significantly reduces cardiac perforation risk compared to procedures without contact force guidance, with particular benefit observed in atrial fibrillation populations [4]. Excessive contact force has been identified as a significant risk factor for serious complications including cardiac perforation, steam pop formation, and thrombus generation during thermal ablation procedures. Although large‐scale pulsed field ablation studies have reported low major complication rates [5], cardiac perforation and tamponade remain potential risks with excessive catheter contact. Therefore, the proposed unipolar electrogram assessment should complement, rather than replace, established safety practices including fluoroscopic guidance, intracardiac echocardiography, and judicious catheter manipulation. Operators must remain vigilant to avoid excessive pressure while pursuing optimal contact, particularly in thin‐walled atrial regions.

The optimal cutoff values for assessing tissue contact based on unipolar positive deflection amplitude and morphology remain undefined. Future prospective studies with larger cohorts should establish quantitative criteria that correlate with effective tissue contact while maintaining procedural safety margins; validate this approach across different catheter designs and clinical scenarios.

## Ethics Statement

The authors have nothing to report.

## Consent

Informed consent was obtained from the patient for participation and publication of this study.

## Conflicts of Interest

The authors declare no conflicts of interest.
